# High co-expression of immune checkpoint receptors PD-1, CTLA-4, LAG-3, TIM-3, and TIGIT on tumor-infiltrating lymphocytes in early-stage breast cancer

**DOI:** 10.1186/s12957-022-02810-z

**Published:** 2022-10-21

**Authors:** Baran Mollavelioglu, Esin Cetin Aktas, Neslihan Cabioglu, Aykhan Abbasov, Semen Onder, Selman Emiroglu, Mustafa Tükenmez, Mahmut Muslumanoglu, Abdullah Igci, Gunnur Deniz, Vahit Ozmen

**Affiliations:** 1 Istanbul Faculty of Medicine, Department of General Surgery, Istanbul University, Istanbul, Turkey; 2 Department of Immunology, Istanbul University, Aziz Sancar Institute of Experimental Medicine, Istanbul, Turkey; 3 Department of Pathology, Istanbul University Istanbul Faculty of Medicine, Istanbul, Turkey

**Keywords:** Early-stage breast cancer, TIL, NK, PD-1, CTLA-4, TIGIT, LAG-3, TIM-3

## Abstract

High expression of immune checkpoint receptors (ICRs) in the tumor microenvironment regulates the anti-tumor response. In this study, the differential expressions of ICRs on tumor-infiltrating lymphocytes (TILs) in patients with early-stage breast cancer were investigated.

The study included 32 patients who underwent surgery with a diagnosis of early-stage breast cancer between September 2018 and March 2020. TIL isolation was performed using a MACS tumor separation device and tumor separation kit. PD-1, CTLA-4, LAG-3, TIM-3, and TIGIT expression of cytotoxic T and natural killer (NK) cells on TILs and peripheral blood lymphocytes (PBLs) were determined by flow cytometry.

Patients with a high Ki-67 index, high TIL density, and HER-2 positivity were more likely to have increased CD16^+^CD56^dim^ NK cells on TILs. Patients with T2 tumors were more likely to have increased expression of PD-1, LAG-3, and TIGIT on tumor-infiltrating CD8^+^ cytotoxic T cells than those with T1 tumors. PD-1, CTLA-4, TIGIT, LAG-3, and TIM-3 expression of CD8^+^ T and CD16^-^CD56^bright^ NK cells in TILs showed significant positive correlations with each other. PD1^+^CD8^+^, TIGIT^+^CD16^+^, and CTLA-4^+^CD56^+^ cells in PBLs and TILs were found to be negatively correlated, whereas only TIM-3^+^ expression of CD8^+^ T and CD16^+^CD56^dim^ cells in PBLs and TILs showed positive correlations.

Our results suggest that CD16^+^CD56^dim^ NK cells on TILs may play a major role in the immune response against HER2-positive or highly proliferating breast tumors in patients with early-stage breast cancer. Furthermore, various ICRs were found to be highly co-expressed with each other on TILs, including PD-1, CTLA-4, LAG-3, TIM-3, and TIGIT. These receptors may synergistically suppress the response to the tumor, which may trigger immune escape mechanisms in the early stage of carcinogenesis. However, ICR expressions other than TIM3 on PBLs were not found to accompany their counterparts on TILs.

## Background

The role of immunotherapy in cancer treatment has been increasing in recent years, and breast cancer is one of the types of cancer in which immunotherapy is widely used. Multiple meta-analyses have demonstrated the prognostic significance of tumor-infiltrating lymphocytes (TILs) in breast cancer, especially in triple-negative and HER-2 positive tumors [1, 2]. ICRs mainly play a role in the immune system’s development of tolerance to its own tissues and in the immune response to the tumor. Many studies have shown that high expression of ICRs in the tumor microenvironment suppresses the immune response to the tumor and causes tumor cells to escape the immune system [3–6].

There have been promising advances in cancer immunotherapy with the blockade of ICRs, such as programmed cell death 1 (PD-1), programmed death-ligand *1* (PD-L1), and cytotoxic T-lymphocyte-associated protein 4 (CTLA-4). As a result, immune checkpoint receptor inhibition has become the backbone of the immunotherapy era. Apart from PD-1, PD-L1, and CTLA-4, studies have also focused on a new generation of target molecules, such as lymphocyte activation gene 3 (LAG-3), T cell immunoglobulin and mucin domain-containing molecule 3 (TIM-3), and T cell immunoreceptor with immunoglobulin (Ig) and immunoreceptor tyrosine-based inhibitory motif (ITIM) domains (TIGIT). PD-1 is an inhibitory receptor that belongs to the CD28 family and is found in many immune cell membranes. The binding of PD-1 to its ligand inhibits the interaction between the major histocompatibility complex (MHC) and T cell receptors (TCR) [7]. The CTLA-4 molecule, like PD-1, is a protein in the CD28 receptor family and is found on the surface of T cells [8] and acts by disrupting B7-CD28 costimulation. CTLA-4 binds to B7 instead of CD28 with a higher affinity, which prevents the immunostimulant effect of B7-CD28 interaction. LAG-3 is a protein found on the surface of T, natural killer (NK), and dendritic cells [9]. The main ligand of LAG-3 is the MHC class II molecule, to which it binds with higher affinity than CD4^+^ T cells. The interaction of LAG-3 with MHC class II exerts an inhibitory effect on regulatory T cells. T cell immunoglobulin and mucin domain-3 (TIM-3) is a surface protein that was first found in CD4^+^ T cells [10]. TIM-3 interacts with galectin-9, which is found on the surface of many tumor cells, and this interaction inhibits the function of helper T and cytotoxic T cells. TIGIT is a member of the immunoglobulin superfamily and is found on the surface of T lymphocytes and NK cells [11]. Studies have been shown that TIGIT inhibits the function of NK and T cells.

Despite all steps taken by immunotherapy, some factors limit its success. The immune response to the tumor varies according to the characteristics of the patient and the tumor. Since many mechanisms work simultaneously in the organism, there is no clear answer to the question of which blockage will be more beneficial in which patient. Therefore, TILs and ICRs, which are the cornerstones of immunotherapy, need to be more firmly connected with clinical practice.

The majority of clinical studies on ICR blockade have included advanced breast cancer patients. Therefore, there is a limited number of studies on the role of TILs and ICRs in early-stage breast cancer and its effect on clinical practice. Alcazar et al. examined the immune escape mechanisms in breast cancer transition from in situ ductal carcinoma to invasive ductal carcinoma. They found that PD-L1 and CTLA-4 levels were significantly higher in cases of invasive ductal carcinoma than in situ ductal carcinoma [12]. These results suggest that ICRs may also play an important role in early-stage breast cancer. In this prospective study, the expression of ICRs on TILs in patients with early-stage breast cancer and their correlations with demographic, pathological, and clinical characteristics were investigated.

## Materials and methods

### Study population

The study included 32 patients who were operated on with a diagnosis of early-stage breast cancer (stages I–II) between September 2018 and March 2020 at the Breast Unit, Department of General Surgery, Istanbul Faculty of Medicine, Istanbul University. Our study was approved by the ethical committee of Istanbul University, Istanbul Medical Faculty, and informed consent was obtained from all patients. Molecular subtypes of the cases were determined according to the preoperative tru-cut biopsy results using immunohistochemical analysis. As in previous studies, patients with luminal A breast cancer were excluded because the immune response is lower in such cases than in other molecular subtypes [13–15]. Surgical materials of eligible patients were evaluated pathologically. The samples were taken from fresh tumor tissue by a breast pathologist and tumor samples were transferred to the flow-cytometry laboratory at the Immunology Department. Pre-operative peripheral blood samples were also taken from six patients. The information recorded included patient age, menopausal status, tumor characteristics (including estrogen and progesterone receptor positivity), HER-2 receptor status, Ki-67 proliferation index, nuclear grade, TIL status, lymphovascular invasion, presence of in situ ductal component, and pathological stages. High TIL density was considered as ≥ 10%.

### Isolation of tumor-infiltrating lymphocytes from tumor tissue

Breast tumor tissues obtained from the surgical specimens were transferred to the Breast Pathology Division. At least 1 cm^3^ of fresh tumoral tissue was transferred to the Immunology Department and preserved in RPMI-1640 medium (Biological Industries, USA) on ice until the TIL isolation procedure. Fresh tumor samples were minced into ~1 mm^3^ fragments and transferred into a gentle MACS C tube containing a mix of enzymes H, R, and A (Tumor Dissociation Kit, human; Miltenyi Biotec, Germany). The single-cell suspension was enriched using the gentleMACS™ dissociator (Miltenyi Biotec, Germany) according to the manufacturer’s protocol. TILs were further purified with Ficoll/Hypaque density gradient centrifugation (Biochrom AG, Berlin, Germany). The PD-1, LAG-3, TIM-3, TIGIT, and CTLA-4 expression of CD8^+^ T lymphocytes and NK cell subsets were analyzed using flow cytometry as described below.

### Immune checkpoint receptor expression of CD8^+^ T cells and NK cell subsets from peripheral blood and tumor tissue

The frequency of immune checkpoint receptors on TIL and peripheral blood of NK and cytotoxic T cells was determined by fluorochrome-labeled mAbs: anti-human CD223 (LAG-3)-FITC, anti-human CD366 (TIM-3)-PerCP/Cy5.5, anti-human CD279 (PD-1)-APC/Cy7, anti-human CD16-AlexaFlour700, anti-human CD56-PECy7, anti-human TIGIT-PE, anti-human CD3-Pacific blue, anti-human CD152 (CTLA-4)-APC, and anti-human CD8aPE/Dazzle monoclonal antibodies (Biolegend, San Diego, CA, USA). The cells were incubated for 30 min at room temperature in the dark. An auto-fluorescent tube was used as an isotypic control for analysis. Following staining, cells were centrifuged with phosphate-buffered saline (PBS) solution once at 2000 rpm for 5 min. The cells were re-suspended with 500 μL of PBS with 1% paraformaldehyde. A FACSAria II flow cytometer (BD Biosciences, San Jose, CA) running FACS Diva software (BD Biosciences, San Jose, CA) was utilized for cell acquisition. FlowJo^TM^10.2 (Tree Star Inc., USA) was used for data analyses for identify the ratio of cytotoxic T cell and NK cell subsets and their immune checkpoint receptor expressions. Peripheral blood and tumor-infiltrating lymphocytes were gated by their size and granularity in the FSC/SSC dot plot histograms. Furthermore, by using the negative and positive gating strategy, CD3-negative and CD3- positive lymphocyte population was identified. The NK cell subsets were further identified on the basis of the expression of CD56 and CD16 as, CD3^-^CD16^+^CD56^dim^ and CD3^-^CD16^-^CD56^bright^ NK cell subsets. CD3^+^CD8^+^ cytotoxic T cells were analyzed by gating on CD3- positive population. Tim-3, LAG-3, PD-1, TIGIT, and CTLA-4 expressions were determined on cytotoxic T cells, and NK cell subsets

### Pathological examination and immunohistochemical analysis

Tumors obtained from the surgical specimens were detail sampled at the Breast Pathology Division. The pathological features of the tumors were evaluated briefly in all cases and included tumor type, tumor size, presence of lymphovascular invasion (LVI), the type and extent of in situ carcinoma, evaluation of TILs, surgical margins, and axillary lymph node status. All cases were routinely assessed for immunohistochemical findings regarding estrogen receptor (ER) (clone SP1, 1:100 dilution; Biocare Concord, CA, USA), progesterone receptor (PR) (clone SP2, 1:400 dilution; Spring Pleasanton, California, USA), human epidermal growth factor receptor-2 (HER-2; clone SP3, 1:200 dilution; Thermo Waltham, Massachusetts, USA), and Ki67 (clone SP6, 1:100 dilution; Biocare Concord, CA, USA). The cutoff value for ER and PR positivity was at least 1% of tumor cells. Immunohistochemical analysis for HER-2 was scored according to the current guidelines of American Society of Clinical Oncology/College of American Pathologists (ASCO/CAP).

### Statistical analysis

Statistical analyses were performed using SPSS version 23 (Statistical Package for Social Sciences; SPSS, Inc., Chicago, IL). Descriptive statistical methods were used in the calculation of the parameters, including the mean and median values with their standard deviations and the minimum and maximum values. The Mann-Whitney *U* test was used to compare two groups of data that did not show normal distribution. Associations of ICR expression with clinicopathologic features of patients were evaluated by Fisher’s exact test. The Spearman test was used for correlation analysis of two groups of data that did not show normal distribution. Statistical significance was accepted as *p* < 0.05 in the analyses.

## Results

### Demographic characteristics of patients

The details of demographic, clinical, and pathological characteristics of the patients are shown in Table 1. The median age of the patients was 50 years (26–68). Of those, 56% were < 50 years old, and 53% were premenopausal. As a surgical procedure, 18 patients (56.3%) underwent mastectomy, and 14 patients (43.7%) underwent breast-conserving surgery. The rates of ER, PR, and HER2 receptor positivity were 78%, 60%, and 33%, respectively. Of those, 73% of patients had T2 tumors, and 27% had pathological axillary lymph node positivity. The LVI rate was 48%, and extensive intraductal component (ductal carcinoma in situ component level > 20%) was 33%. High TIL density (> %10) was observed in 12 patients (37.5%) (Table 1).

**Table 1:** Demographic, pathological features and surgical interventions

	***n***	%
**Age (year)**		
*Median (min–max)*	49 (26–68)	
*Mean ± SD*	50.27 ± 10.83	
**≤ 50**	18	56.3
**> 50**	14	43.7
**Menopausal status**		
**Premenopausal**	17	53.2
**Postmenopausal**	15	46.8
**Surgical interventions**		
**Simple mastectomy**	5	15.6
**Areola sparing mastectomy**	12	37.5
**Skin sparing mastectomy**	1	3.1
**Breast conserving surgery**	14	43.8
**Axillary approach**		
**SLNB (−)**	24	75
**SLNB (+)**	5	15.7
**ALND**	3	9.3
**HER-2**		
**Negative**	21	65.7
**Positive**	11	34.3
**ER**		
**Negative**	7	21.8
**Positive**	25	78.2
**PR**		
**Negative**	12	37.5
**Positive**	20	62.5
**Ki-67 (%)**		
*Median (Min–Max)*	32.5 (12–90)	
*Mean ± SD*	38.9 ± 18.8	
**Ki-67 ≤ 20**	5	15.6
**Ki-67 > 20**	27	84.4
**Histological grade**		
**Grade 2**	10	31.2
**Grade 3**	22	68.8
**High TIL density (>10%)**		
**Negative**	20	62.5
**Positive**	12	37.5
**Receptor status**		
**Luminal B**	26	81.2
**HER2 positive**	3	9.4
**Triple negative**	3	9.4
**Luminal**		
**Negative**	6	18.7
**Positive**	26	81.3
**T stage**		
**T1**	9	28.1
**T2**	23	71.9
**N stage**		
**N0**	23	71.9
**N1**	9	28.1
**Stage**		
**Stage 1**	9	28.1
**Stage 2**	23	71.9
**Lymphovascular invasion**		
**Negative**	16	50
**Positive**	16	50
**Extensive intraductal component**		
**Negative (< 20%)**	21	65.7
**Positive (> 20%)**	11	34.3

### Immune checkpoint receptor expression on TILs

**Fig. 1:**
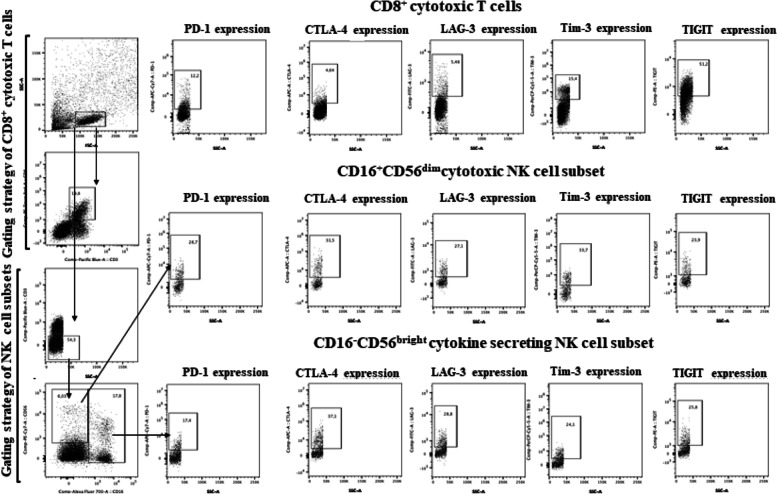
Flow cytometric analysis of TILs subsets. A representative example of the flow cytometric gating strategy used for the immune checkpoint receptor analysis of NK cell subsets and cytotoxic T cells is shown. Tumor infiltrated lymphocytes were identified by their size and granularity in the FSC/SSC dot plot, and NK cells were gated on the SSC/CD3- and then gated further as CD56^bright^ or CD56^dim^ NK cells. Cytotoxic T cells were identified by gating on CD3^+^/CD8^+^ quadrant. PD-1, TIGIT, CTLA-4, LAG-3, and Tim-3 expressions were evaluated on CD8^+^ T cells and cytotoxic (CD16^+^CD56^dim^) and cytokine secreting (CD16^-^CD56^bright^) NK cells. PD-1, TIGIT, CTLA-4, LAG-3, and Tim-3 expressions are given as percentage of the designated population. Data were analyzed using the FlowJo^TM^10.2 software

**Fig. 2:**
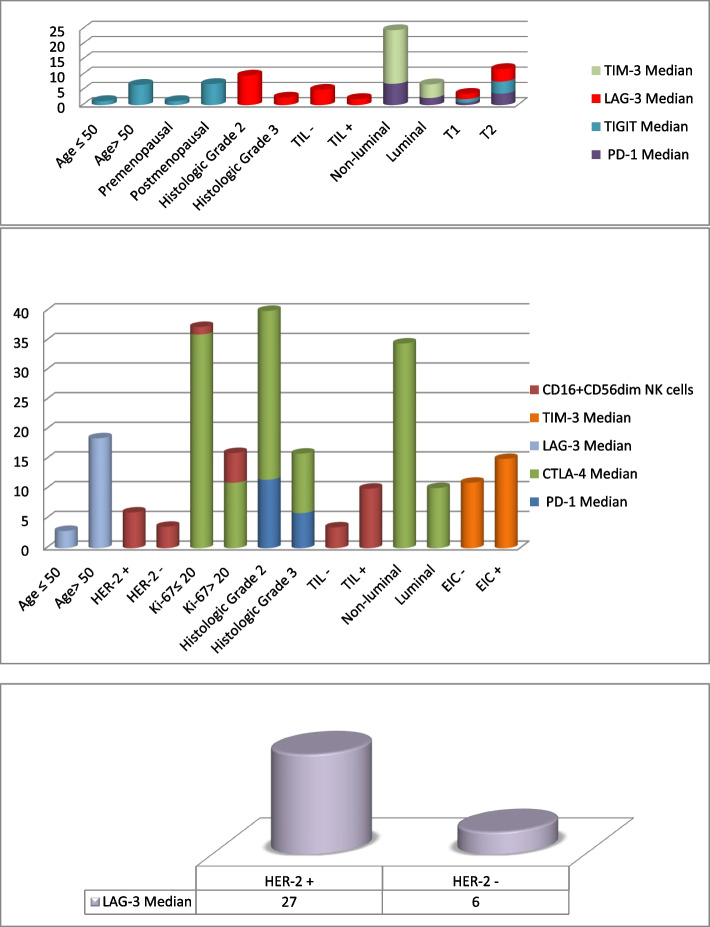
Demographic, clinical, and pathological features and ICR expressions on tumor-infiltrating CD8^+^ T cells (**a**), on tumor-infiltrating CD16^+^CD56^dim^ NK cell subset (**b**), and LAG-3 expression on tumor infiltrating CD16^-^CD56^bright^ NK cell subset (**c**)

**Fig. 3:**
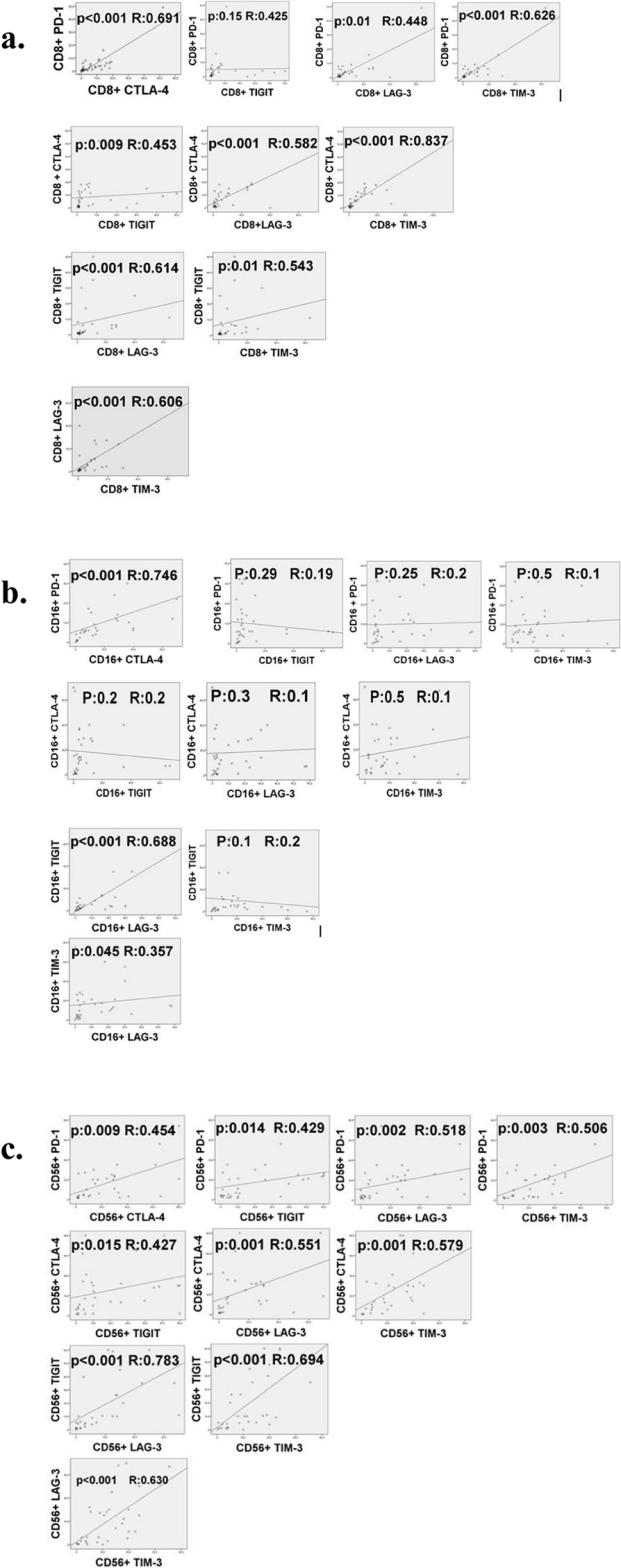
Correlation of immune checkpoint receptors (ICR) on tumor-infiltrating CD8^+^ TILs (**a**), correlation of ICR on tumor-infiltrating CD16^+^CD56^dim^ NK cells (**b**), and correlation of ICR on tumor-infiltrating CD16^-^CD56^bright^ NK cells (**c**)

Expression of ICR were separately evaluated on CD8^+^ T lymphocytes and cytotoxic (CD16^+^CD56^dim^) and cytokine secreting (CD16^-^CD56^bright^) NK cells in both TILs and PBLs (Fig. 1). The associations between ICR expression on TILs and demographic, clinical, and pathological features were examined (Fig. 2). Statistically significant positive correlations were found between ICR expressions including PD-1, CTLA-4, TIGIT, LAG-3, and TIM-3 on CD8+ T cells (PD-1 and CTLA-4, PD-1 and LAG-3, PD-1 and TIM-3, CTLA-4 and TIGIT, CTLA-4 and LAG-3, CTLA-4 and TIM-3, TIGIT and LAG-3, TIGIT and TIM-3, LAG-3 and TIM-3) except the correlation between PD-1 and TIGIT (*p* = 0.15) (Fig. 3a).

PD-1 was positively correlated with CTLA-4, whereas LAG-3 was positively correlated with TIGIT and TIM-3 on CD16^+^CD56^dim^ NK cells (*R* = 0.74, *p* < 0.001; *R* = 0.68, *p* < 0.001; *R* = 0.35, *p* = 0.045, respectively) (Fig. 3b). Expression of PD-1, CTLA-4, TIGIT, LAG-3, and TIM-3 on CD16^-^CD56^bright^ NK cells were positively correlated with each other (Fig. 3c). However, PD1^+^CD8^+^, TIGIT^+^CD16^+^ and CTLA-4^+^CD56^+^ cells both in PBLs and TILs were negatively correlated(*R* = − 0.98 *p* < 0.001; *R* = − 0.84 *p* = 0.03; *R* = − 0.81 *p* = 0.05, respectively). Interestingly, only TIM-3 expression on CD8^+^ and CD16^+^CD56^dim^ NK cells in PBLs and TILs showed positive correlations, but these associations did not reach statistical significance (*p* = 0.086 and *p* = 0.072, respectively) (Table 2).

**Table 2:** Significant correlations of immune checkpoint receptors (ICR) on TILs and PBLs

		PBL PD-1^**+**^ CD8^**+**^	PBL TIM-3^**+**^ CD8^**+**^	PBL TIGIT^**+**^ CD16^**+**^	PBL TIM-3^**+**^ CD16^**+**^	PBL CTLA-4^**+**^ CD56^**+**^
**TIL** **PD-1**^**+**^**CD8**^**+**^	*R*	**− 0.98**	0.05	− 0.75	0.55	− 0.72
	*p*	**0.0001**	0.91	0.08	0.25	0.10
**TIL** **TIM-3**^**+**^**CD8**^**+**^	*R*	− 0.46	**0.75**	− 0.31	0.81	− 0.11
	*p*	0.35	**0.08**	0.53	0.05	0.82
**TIL** **TIGIT**^**+**^**CD16**^**+**^	*R*	− 0.75	0.26	**− 0.84**	0.46	− 0.72
	*p*	0.08	0.61	**0.03**	0.35	0.10
**TIL** **TIM-3**^**+**^**CD16**^**+**^	*R*	− 0.37	0.63	− 0.25	**0.77**	− 0.25
	*p*	0.46	0.17	0.62	**0.07**	0.62
**TIL** **CTLA-4**^**+**^**CD56**^**+**^	*R*	− 0.81	− 0.27	− 0.84	0.11	**− 0.81**
	*p*	0.05	0.59	0.03	0.82	**0.05**

Patients >50 years old were found to have higher levels of LAG-3 expression on CD16^+^CD56^dim^ NK cells and TIGIT expression on CD8^+^ cytotoxic T cells (*p* = 0.023 and *p* = 0.08, respectively). Patients with T2 tumors were more likely to express PD-1 (*p* = 0.020), TIGIT (*p* = 0.097), and LAG-3 (*p* = 0.098) on CD8^+^ T cells on TILs than those with T1 tumors. In contrast, PD-1^+^ and CTLA-4^+^CD16^+^CD56^dim^ NK cells and LAG-3^+^CD8^+^ T cells were found to be increased in patients with histologic grade 2 (HG2) tumors than those with HG3 tumors (*p* = 0.038, *p* = 0.022, and *p* =0.077 respectively).

Similarly, PD-1^+^CD8^+^ and TIM-3^+^CD8^+^ T cells and CTLA-4^+^CD16^+^CD56^dim^ NK cell subsets on TILs were found to be elevated in patients with non-luminal breast cancer, but these associations did not reach statistical significance either (*p* = 0.074, *p* = 0.062, and *p* = 0.063, respectively). The CD16^+^CD56^dim^ NK cell ratio was found to be significantly increased in patients with a high Ki-67 index (> 20%) (*p* = 0.035) and high TIL density (*p* = 0.002). Furthermore, patients with HER-2 positivity were more likely to have an increased CD16^+^CD56^dim^ NK cell ratio, but the difference did not reach statistical significance (*p* = 0.07) (Tables 3, 4, and 5).

**Table 3:** Demographic, clinical, and pathological features and ICR expressions on tumor-infiltrating CD8+ T cells

	PD-1^**+**^CD8^+^ T cells		CTLA-4^**+**^CD8^+^ T cells		TIGIT^**+**^ CD8^+^ T cells		LAG-3^**+**^ CD8^+^ T cells		TIM-3^**+**^ CD8^+^ T cells	
	Median (min–max)	***p***	Median (min–max)	***p***	Median (min–max)	***p***	Median (min-max)	***p***	Median (min–max)	***p***
**Age**										
**≤ 50(*n*: 18)**	2.1 (0.3–49)	0.97	5.5 (0.2–52)	0.64	1.3 (0.6–11)	**0.08**	2.4 (0.5–64)	0.28	3.4 (0.2–63)	0.89
**> 50** **(*n:*14)**	10.5 (1.3–42)		7.5 (0–18)		6.6 (0.2–50)		7.5 (0.2–40)		7.4 (0.3–30)	
**Menopausal status**										
**Pre** **(*n:*17)**	2 (0.3–49)	0.86	6 (0.2–52)	0.40	1.3 (0.6–11)	**0.04**	2.8 (0.6–64)	0.88	4 (0.2–63)	0.69
**Post(*n:*15)**	3.8 (0–9)		6 (0–16)		6.9 (0.2–50)		5.5 (0.2–40)		6 (0.3–30)	
**HER-2**										
**Negative (*n:*21)**	2.7 (0–16)	0.76	6 (0–19)	0.53	1.5 (0,6–43)	0.37	3.8 (0.2–40)	0.63	4 (0.3–27)	0.18
**Positive (*n:*11)**	3 (0.3–49)		6 (0.3–52)		6 (0.2–50)		4 (0.6–64)		11 (0.2–63)	
**Ki-67**										
**≤ 20 (*n:*5)**	0.5 (0–49)	0.55	4 (0–52)	0.62	6.9 (0.2–25)	0.60	10.4 (1.2–64)	0.26	1.2 (1–63)	0.77
**> 20 (*n:*27)**	3 (0.3–16)		6 (0.2–19)		2 (0.6–50)		3.8 (0.2–27)		5 (0.2–30)	
**Histologic grade**										
**Grade 2 (*n:*10)**	2.5 (0–49)	0.72	7.5 (0–52)	0.26	4.1 (0.9–43)	0.39	9.7 (0.8–64)	**0.07**	6.8 (1–63)	0.62
**Grade 3 (*n:*22)**	3 (0.3–16)		4.6 (0.2–18)		2 (0.2–50)		2.5 (0.2–27)		3.9 (0.2–30)	
**High TIL density (>%10)**										
**Negative (*n:*20)**	2.9 (0–49)	0.81	8 (0–52)	0.43	2.2 (0.2–43)	0.42	5 (1–64)	**0.02**	4.5 (0.4–63)	0.92
**Positive (*n:*12)**	2.6 (0.4–8)		4.1 (0.6–15)		3.7 (0.9–50)		2 (02–11)		5.5 (0.2–30)	
**Luminal**										
**Negative (*n:*6)**	7 (1–49)	**0.07**	9 (2.7–52)	0.19	6.5 (1.3–30)	0.12	3.4 (0.5–64)	0.71	17.6 (1–63)	**0.06**
**Positive (*n:*26)**	2.4 (0–16)		5.5 (0–19)		1.4 (0.2–50)		4 (0.2–40)		4.4 (0.2–27)	
**T stage**										
**T1 (*n:*9)**	0.9 (0.3–6.1)	**0.02**	4 (0.2–18)	0.41	1.1 (0.2–17)	**0.09**	1.7 (0.2–27)	**0.09**	1.2 (0.2–19)	0.33
**T2 (*n:*23)**	3.8 (0–49)		7 (0–52)		4 (0.6–50)		4 (0.5–64)		5 (0.4–63)	
**N stage**										
**N0 (*n:*23)**	2 (0–49)	0.41	6 (0–52)	0.78	2.4 (0.2–50)	0.80	3.8 (0.2–64)	0.98	6 (0.2–63)	0.73
**N1 (*n:*9)**	3 (0.5–9)		6 (1.1–16)		1.5 (0.6–35)		4 (0.8–24)		4.9 (1.5–12)	
**Lympho-vascular invasion**										
**Negative (*n:*16)**	2.5 (0–8)	0.33	3 (0–19)	0.11	1.8 (0.2–43)	0.70	2.9 (0.2–40)	0.20	3.4 (0.2–30)	0.16
**Positive (*n:*16)**	3.4 (0.3–49)		10 (0.2–52)		2.2 (0.6–50)		4.2 (1–64)		7.5 (0.7–63)	
**Extensive intraductal component**										
**Negative (*n:*21)**	2.2 (0–8)	0.2	6 (0–19)	0.45	1.3 (0.2–43)	0.23	3.8 (0.8–40)	0.84	4 (0.4–19)	0.21
**Positive (*n:*11)**	3.8 (0.4–49)		11 (0.6–52)		5 (0.6–50)		5.5 (0.2–64)		9 (0.2–63)

**Table 4:** Demographic, clinical, and pathological features and ICR expressions on tumor-infiltrating CD16^+^CD56^dim^ NK cell subset

	CD16^**+**^CD56^**dim**^ NK cells		PD-1^**+**^ NK cells		CTLA-4^**+**^ NK cells		TIGIT^**+**^ NK cells		LAG-3^**+**^ NK cells		TIM-3^**+**^ NK cells	
	Median (min–max)	***p***	Median (min–max)	***p***	Median (min–max)	***p***	Median (min–max)	***p***	Median (min–max)	***p***	Median (min–max)	***p***
**Age**												
**≤ 50** **(*n:*18)**	4 (0.5–15)	0.38	6.6 (0–32)	0.93	14.5 (0–67)	0.73	3.5 (0–14)	0.33	2.9 (0–24)	**0.02**	13 (1.3–75)	0.92
**> 50** **(*n:*14)**	5.5 (1–37)		5.8 (0.8–30)		10 (0.5–70)		4 (0–67)		18.5 (0–58)		13.5 (0–55)	
**Menopausal status**												
**Pre** **(*n:*17)**	4.6 (0.5–15)	0.98	7 (0–32)	0.97	15 (0–67)	0.74	3,6 (0–35)	0.49	3 (0–34)	0.22	15 (1.3–75)	0.86
**Post** **(*n:*15)**	5 (1–37)		6 (0.8–30)		11 (0.5–70)		4 (0–67)		5.3 (0–58)		13 (0–55)	
**HER-2**												
**Negative (*n:*21)**	3.6 (0.5–12)	**0.07**	7.2 (0–32)	0.35	14 (0–70)	0.29	4 (0–64)	0.78	3 (0–58)	0.27	15 (0–75)	0.59
**Positive (*n:*11)**	6 (1.3–37)		5.5 (0.4–24)		6.9 (0.5–67)		2.8 (1–67)		16 (0.8–57)		8 (1.3–60)	
**Ki-67**												
**≤ 20 (*n:*5)**	1.3 (1–4)	**0.03**	12 (4–30)	0.35	36 (3.6–70)	**0.07**	2.8 (0–4)	0.14	18 (0–30)	0.91	40 (0–60)	0.51
**> 20 (*n:*27)**	5 (0.5–37)		6 (0–32)		11 (0–40)		4 (0–67)		3.4 (0–58)		13 (0.6–75)	
**Histologic grade**												
**Grade 2 (*n:*10)**	3.7 (1–11)	0.17	11.5 (4.8–32)	**0.03**	28.5 (3.6–70)	**0.02**	4 (0–35)	0.80	15 (0–30)	0.52	18 (0–60)	0.32
**Grade 3 (*n:*22)**	5 (0.5–37)		5.9 (0–32)		10 (0–40)		3.7 (0–60)		3 (0–58)		9.3 (0.6–75)	
**High TIL density (>%10)**												
**Negative (*n:*20)**	3.5 (0.5–11)	**0.002**	8.1 (0–32)	0.22	14 (0–70)	0.15	4 (0–35)	0.69	11 (0–34)	0.54	15.5 (0–75)	0.37
**Positive (*n:*12)**	10 (1.5–37)		5.9 (0.4–24)		7 (0.5–40)		2.7 (0.4–67)		3 (1–58)		8.7 (1.3–28)	
**Luminal**												
**Negative (*n:*6)**	5 (2–37)	0.28	6.5 (4–14)	0.82	34.5 (0.5–67)	**0.06**	4.5 (0.4–35)	0.86	2.8 (1.1–34)	0.62	17.7 (3–60)	0.38
**Positive (*n:*26)**	4.3 (0.5–15)		6 (0–32)		10.1 (0–70)		4 (0–67)		4.8 (0–58)		13.5 (0–75)	
**T stage**												
**T1 (*n:*9)**	1.5 (0.5–15)	0.28	6 (0.4–24)	0.62	9 (1–40)	055	4 (1–35)	0.40	16 (0.8–34)	0.40	8 (1.6–40)	0.95
**T2 (*n:*23)**	5 (1–37)		6 (0–32)		13 (0–70)		4 (0–67)		3 (0–58)		14 (0–75)	
**N stage**												
**N0 (*n:*23)**	5 (0.5–37)	0.8	6 (0–24)	0.11	9.2 (0–70)	0.52	3.4 (0–67)	0.45	3.4 (0–57)	0.39	11 (0–75)	0.73
**N1 (*n:*9)**	4.6 (1–11)		10 (1–32)		14 (1–37)		4 (2–64)		5.3 (1.8–58)		15 (1.3–55)	
**Lympho-vascular invasion**												
**Negative (*n:*16)**	5.7 (1–37)	0.3	6 (0.4–32)	0.67	9.1 (0.5–70)	0.49	3.5 (0–64)	0.52	3.2 (0–58)	0.66	12 (0–40)	0.46
**Positive (*n:*16)**	4 (0.5–12)		7.1 (0–32)		14.5 (0–67)		4.5 (0–67)		10.7 (0–57)		14.8 (1.6–75)	
**Extensive intraductal component**												
**Negative (*n:*21)**	5 (0.5–12)	0.13	6 (0.8–32)	0.84	12 (1–70)	0.93	4 (0–64)	0.75	5.3 (0–58)	1	11 (0–40)	**0.08**
**Positive (*n:*11)**	3.6 (1–37)		7 (0–30)		13 (0–67)		3.4 (0–67)		3 (0–57)		15 (3–75)

**Table 5:** Demographic, clinical, and pathological features and ICR expressions on tumor-infiltrating CD16^-^CD56^bright^ NK cell subset

	PD-1^**+**^ NK cells		CTLA-4^**+**^ NK cells		TIGIT^**+**^ NK cells		LAG-3^**+**^ NK cells		TIM-3^**+**^ NK cells	
	Median (min–max)	***p***	Median (min–max)	***p***	Median (min–max)	***p***	Median (min–max)	***p***	Median (min–max)	***p***
**Age**										
**≤ 50 (*n:*18)**	13 (1.5–74)	0.7	16 (1.8–80)	0.95	7.5 (1–60)	0.64	6.1 (0.1–67)	0.38	21 (1–71)	0.83
**> 50 (*n:*14)**	15.5 (2–35)		23.5 (1.3–80)		10.5 (0–60)		25 (0–70)		22 (0.4–48)	
**Menopausal status**										
**Pre** **(*n:*17)**	20 (1,5–74)	0.97	24 (1.8– 80)	0.28	9 (1.2–60)	0.86	11 (0,1–68)	0.86	24 (1–71)	0.97
**Post** **(*n:*15)**	11 (2–35)		15 (1.3–62)		10 (0–60)		22 (0–70)		21 (0.4–48)	
**HER-2**										
**Negative (*n:*21)**	11 (1.5–74)	0.49	15 (1.3–80)	0.17	6 (0–60)	0.30	6 (0–50)	**0.05**	20 (0.4–48)	0.14
**Positive (*n:*11)**	20 (1.7–56)		30 (1.8–80)		11 (2–59)		27 (0.3–70)		32 (1–71)	
**Ki-67**										
**≤ 20 (*n:*5)**	6 (1.7–56)	0.58	18 (1.8–65)	0.83	10 (0–35)	0.67	25 (0–70)	0.83	21 (1–71)	0.69
**> 20 (*n:*27)**	20 (1.5–74)		17 (1.3–80)		10 (0.3–60)		11 (0.1–68)		23 (0.4–48)	
**Histologic grade**										
**Grade 2 (*n:*10)**	15.5 (1.7–74)	0.90	29.5 (1.8–80)	0.10	8 (0–48)	0.77	19.5 (0–67)	0.72	24.5 (1–71)	0.45
**Grade 3 (*n:*22)**	13 (1.5–35)		13.5 (1.3–80)		10 (0.3–60)		11 (0.1–70)		17 (0,4–48)	
**High TIL density (>%10)**										
**Negative (*n:*20)**	15.5 (1.5–74)	0.96	21 (1.5–80)	0.93	7.7 (0–60)	0.47	12.5 (0–70)	0.74	23 (0.4–71)	0.95
**Positive (*n:*12)**	13 (2–35)		14.4 (1.3–41)		15 (1.2–60)		16.5 (0.1–32)		20.5 (1.7–48)	
**Luminal**										
**Negative (*n:*6)**	13 (2–56)	0.80	24.5 (8.3–80)	0.25	22.5 (1.2–51)	0.61	17 (0.1–68)	0.59	27.5 (4–71)	0.56
**Positive (*n:*26)**	15.5 (1.5–74)		17.5 (1.3–80)		9.5 (0–60)		12.5 (0–70)		20.5 (0.4–48)	
**T stage**										
**T1 (*n:*9)**	6 (1.7–35)	0.45	13.1 (1.3–80)	0.60	26 (1.3–60)	0.26	28 (0.3–70)	0.12	18 (1–48)	0.58
**T2 (*n:*23)**	16 (1.5–74)		24 (1.5–80)		9 (0–60)		11 (0–67)		23 (0.4–71)	
**N stage**										
**N0 (*n:*23)**	10 (1.5–74)	0.34	13.8 (1.3–80)	0.27	11 (0–60)	0.45	25 (0–70)	0.35	20 (0.4–71)	0.58
**N1 (*n:*9)**	20 (3.2–35)		30 (6–62)		6 (1.3–60)		11 (1.5–25)		24 (3.4–45)	
**Lympho-vascular invasion**										
**Negative (*n:*16)**	6.5 (2–74)	0.28	16 (1.3–80)	0.47	7.5 (0–60)	0.45	8.6 (0–70)	0.48	23.5 (0.4–45)	0.92
**Positive (*n:*16)**	21 (1.5–56)		27 (1.8–80)		10.2 (1.2–60)		21.5 (0.1–68)		18.5 (1–71)	
**Extensive intraductal component**										
**Negative (*n:*21)**	10 (1.7–74)	0.63	18 (1.5–80)	0.73	5.4 (0–60)	0.24	11 (0–70)	0.55	20 (0.4–48)	0.42
**Positive (*n:*11)**	16 (1.5–56)		15 (1.3–80)		10.3 (1–59)		25 (0.4–68)		23 (1.7–71)	

## Discussion

High expression of ICRs in the tumor microenvironment suppresses the immune response against the tumor and causes the tumor cells to evade the immune system. The number of studies on ICRs has been increasing in recent years, and it has been shown that their blockages increase the effectiveness of cancer treatment [16]. Determining the associations of clinical, pathological, and demographic characterizations with ICRs may contribute to the optimization of ICR-targeted treatments.

The success of immunotherapy, which includes targeted monoclonal antibody treatments, is directly related to the high expression of ICRs in cancerous tissue. Sasidharan et al. showed that genes expressing PD-1, CTLA-4, TIM-3, and LAG-3 in breast cancer patients had higher levels in breast cancer tissue than in normal breast tissue [17]. It has also been examined the levels and prognostic values of 50 ICR genes in molecular subgroups of breast cancer, including PD-1, CTLA-4, TIGIT, LAG-3, and TIM-3 [18]. The results demonstrated that the levels were higher in breast cancer patients than healthy subjects.

There have been only a few reports on ICR expression of PBLs in breast cancer patients. Elashi et al. found that transcriptomic expressions of PD-1, CTLA-4, TIM-3, TIGIT, and PDL-1 were upregulated in the peripheral blood of primary breast cancer patients, but LAG-3 expression was downregulated [19].

In our study, no significant association was found between PD-1, CTLA-4, TIM-3, LAG-3, and TIGIT expression on PBLs and clinicopathological data. However, a negative correlation was observed between PD1^+^CD8^+^, TIGIT^+^CD16^+^, and CTLA-4^+^CD56^+^ cells in PBLs and their counterparts in TILs. This negative correlation may indicate a decrease in tumor-specific ICR expressing PBLs through migration of these lymphocytes from the blood to the tumor area, which may occur through specific chemokine receptor and chemokine interactions between immune cells and the tumor in tumor-associated stroma [20, 21]. However, TIM-3 expression of CD8^+^ and CD16^+^CD56^dim^ cells in PBLs and TILs has shown positive correlations, but the results did not reach statistical significance. The discordance between ICR expressions on PBLs and TILs might be due to the limited numbers of PBL samples. Therefore, more studies are warranted to make an accurate conclusion on this subject [22, 23].

In the era of the immune checkpoint blockade, PD1^+^CD8^+^ cells in PBL are also being investigated as a promising biomarker to monitor the effectiveness of anti-PD-1 and PDL-1 treatments [24]. Kamphorst et al. demonstrated an increase of PD1^+^CD8^+^ cells in PBLs in patients diagnosed with advanced lung cancer and showed clinical benefit following PD-1-targeted therapy [25]. Based on these contrary findings, more studies are needed to examine the role of ICR-expressing PBLs and their interactions with ICR-expressing TILs in cancer treatment.

Lei Tu et al. showed that high expression of PD-1, CTLA-4, LAG-3, and TIM-3 is a good prognostic marker in patients with breast cancer who received chemotherapy, and higher ICRs expressions were detected on TILs of these patients [26]. The presence of high TIL levels was found as a marker of the immune response of the organism to tumor cells, which seems to be associated with increased survival and good prognosis. In a meta-analysis, Mao et al. found an increased percentage of TILs to be a good prognostic factor for triple-negative tumors [1]. Furthermore, a high level of tumor-infiltrating CD8^+^ cytotoxic T lymphocytes was associated with prolonged survival and disease-free survival for patients with triple-negative and hormone receptor-positive molecular subtypes. In another meta-analysis, it has been shown that high TIL levels were associated with improved survival in patients with triple-negative breast cancer [2].

The prognostic significance of PD-L1 and high TIL expression has been investigated in breast cancer in many studies [27–30]. In most of these reports, it was concluded that higher expression of PD-L1 was associated with poor prognosis in patients with aggressive tumor characteristics. Okabe et al. investigated the expression of PD-1/PD-L1 and the density of CD8^+^ and CD3^+^ lymphocytes in tumor tissue by immunohistochemical analysis [27]. The study included 97 patients with early-stage breast cancer, and it was revealed that those with high CD8^+^/PD-L1^+^ TIL levels had lower survival rates. In a meta-analysis by Zhang et al., high PD-L1 level was similarly found to be associated with decreased overall survival, lymph node positivity, high HG, hormone receptor negativity, and the triple-negative molecular subtype of breast cancer [28]. PD-L1 positivity was found to be associated with a poor outcome in a meta-analysis by Wang et al., which included 8583 patients, and one by Kim et al., which included 7877 patients [29, 30].

Patients older than 50 were more likely to have increased LAG-3 expression on CD16+CD56dim NK cells and increased TIGIT expression on CD8+ T cells on TILs. In concordance with these findings, Zhang et al. reported higher TIM-3 expressions in patients over 45 years of age in their study [31]. However, there are also many studies reporting higher ICRs expression on TILs in younger patients [32–34]. Furthermore, in our cohort, patients with larger tumor size were more likely to have LAG-3 expression on CD8^+^ T cells, but the results did not reach statistical significance (*p* = 0.09). Burugu et al. also showed that LAG-3 expression is associated with large tumor size [35].

HER-2 positive and triple-negative molecular subtypes of breast cancer are known to be more immunogenic tumor types and have higher TIL rates than luminal subtypes [13, 14]. NK cells are important effector cells against tumors, and increasing their activity will strengthen the immune response. Unlike T cells, NK cells have MHC-independent cytotoxicity, but the number and activity of NK cells are generally suppressed by immunosuppressive factors in the tumor microenvironment [36]. Immune checkpoint receptors are one of these factors. As a result, immunotherapy modalities targeting NK cells, like immune checkpoint blockade, have become a rising star in cancer treatment in recent years [20, 21].

In the present study, we have demonstrated significantly elevated tumor-infiltrating cytotoxic CD16^+^CD56^dim^ NK cell rates in patients with a high TIL ratio and high Ki-67 proliferation index. Moreover, a trend of increased CD16^+^CD56^dim^ NK and LAG-3-expressing CD16^-^CD56^bright^ NK cell subsets in patients with HER2 positivity were observed. These findings suggest that NK cells may play an important role in the immune response against HER2-positive or highly proliferating tumors, which is concordant with previous reports [37, 38]. Furthermore, these findings also indicate that patients with a high Ki67 index and HER2 positivity might be suitable candidates for NK targeted immune checkpoint blockade.

Kim et al. showed that better therapeutic effects of chemotherapy were significantly associated with HER-2 positivity and higher NK cells in patients with breast cancer. Study examined the expression of PD-1, PD-L1, PD-L2, CTLA-4, LAG-3, and TIM-3 in breast cancer patients by flow cytometry and immunohistochemical analysis and showed that ICR expressions were higher in triple-negative and HER-2 positive patients, and expression levels differed significantly even in tumors in the same molecular subgroup [39].

In concordance with previously published reports, our results showed that PD-1 and TIM-3 expression on CD8^+^ T cells and CTLA-4 expression on CD16^+^CD56^dim^ NK cells were higher in patients with non-luminal tumors [32, 34]. Nevertheless, more studies are needed to evaluate the ICR expression on CD16^+^CD56^dim^ NK cells and the effectiveness of anti-ICR treatment in patients with luminal-type tumors.

Furthermore, our results also revealed that CD16^+^CD56^dim^ NK cells and LAG-3 expressing CD8^+^ T cells on TILs were significantly higher in patients with high TIL density. Similarly, other studies also demonstrated high expression of ICRs in patients with high TIL levels [26, 40]. Baitsch et al. reported that in patients with malignant melanoma, tumor-infiltrating CD8^+^ T cells express many inhibitory molecules, such as BTLA, TIM-3, LAG-3, KRLG-1, 2B4, CD160, PD-1, and CTLA-4 [41]. Cabioglu et al. analyzed the expressions of PDL-1, PD-1, TIM-3, LAG-3, and CTLA-4 immunohistochemically in surgical specimens from patients with locally advanced triple-negative breast cancer after neoadjuvant chemotherapy and showed that PD-1, PDL-1, CTLA-4, TIM-3 and LAG-3 in tumors and TILs were positively correlated with each other [42].

We recently reported that the new generation ICRs TIM-3, LAG-3, and TIGIT are highly expressed in locally advanced breast cancer with poor prognostic factors following neoadjuvant chemotherapy [43]. Byun et al. also found that high TIM-3 expression on TILs was associated with high PD-1 and PD-L1 expression [33]. Furthermore, Burugu et al. showed that there is a high rate of PD-1 expression on LAG-3^+^ TILs [35]. In another study, high TIM-3 levels on TIL were related to high PD-1 and high LAG-3 levels on TILs [34]. We similarly found that PD-1, CTLA-4, TIM-3, LAG-3, and TIGIT expressions on CD8^+^ T and CD16^-^CD56^bright^ NK cells on TILs had high correlation with each other. These findings may indicate that patients who would benefit more from various immunotherapies can be identified by evaluating one of the ICRs, such as PD-1 and PD-L1, which are the most frequently used predictive markers in the immunotherapy era in clinical practice. In this study, we used flow cytometric analysis to investigate the expression of ICR on TILs. Therefore, all these findings in the present study should be validated by using IHC technique or other molecular assays like RT-PCR in future. Furthermore, due to the early stage nature of our patients with limited follow up data, we plan to investigate the prognostic significance of different ICR expressions on TILs with longer follow-up in future. More studies are needed to confirm these results.

In conclusion, ICRs on cytotoxic T cells and on CD16^-^CD56^bright^ NK cells were found to be highly co-expressed with each other, including PD-1, CTLA-4, LAG-3, TIM-3, and TIGIT. These receptors may synergistically suppress the response to the tumor, which may trigger immune escape mechanisms in the early stage of carcinogenesis. The high co-expression of ICRs, which may have a similar and synergistic effect in the tumor microenvironment, suggests that combined immunotherapy options could improve the success of cancer treatment. Furthermore, NK cells may play an important role in the immune response against HER2-positive or highly proliferating tumors. This suggest that patients with a high Ki67 index and HER2 positivity may be more likely to benefit from NK-targeted immune checkpoint blockade strategies that should be investigated in future studies.

## Data Availability

The datasets during and/or analyzed during the current study are available from the corresponding author on reasonable request.
